# Pseudomonas aeruginosa in Ventilator-Associated Pneumonia: A Comprehensive Review of Resistance and Clinical Outcomes

**DOI:** 10.7759/cureus.108901

**Published:** 2026-05-15

**Authors:** Sneha D Parit, Priyanka M Mane, Satish R Patil

**Affiliations:** 1 Department of Microbiology, Krishna Vishwa Vidyapeeth (Deemed to be University), Karad, IND

**Keywords:** antimicrobial resistance, biofilm formation, esbl, intensive care unit (icu), metallo-beta-lactamase, multidrug resistance, pseudomonas aeruginosa, ventilator-associated pneumonia (vap)

## Abstract

Ventilator-associated pneumonia (VAP) remains one of the most common and serious healthcare-associated infections in intensive care units (ICUs), contributing significantly to patient morbidity and mortality among patients on mechanical ventilation. Due to its virulence and increasing antimicrobial resistance, *Pseudomonas aeruginosa* poses a significant clinical challenge. This review provides a comprehensive analysis of the epidemiology, pathogenesis, resistance mechanisms, and clinical outcomes associated with *P. aeruginosa*-induced VAP. Among the most notable virulence mechanisms are quorum sensing, biofilm formation, type III secretion systems, and host immune defence resistance. Current therapeutic options, such as piperacillin-tazobactam, meropenem, and newer β-lactam/β-lactamase inhibitor combinations, are reviewed in the context of emerging resistance. Preventive strategies such as antimicrobial-coated endotracheal tubes, cuff pressure adjustment, and subglottic secretion drainage are also discussed. The rising incidence of multidrug-resistant *P*. *aeruginosa* indicates the critical requirement for enhanced antimicrobial stewardship and novel therapeutic approaches to lessen the impact of VAP in critically ill patients. Additionally, this review addresses intrinsic, acquired, and adaptive resistance mechanisms, the clinical burden associated with multidrug-resistant and extensively drug-resistant strains, including increased mortality and prolonged ICU stay, advancements in rapid diagnostic techniques such as molecular assays and matrix-assisted laser desorption ionization-time of flight mass spectrometry (MALDI-TOF MS), and emerging therapeutic strategies, including cefiderocol, bacteriophage therapy, and anti-biofilm approaches.

## Introduction and background

Healthcare-associated infections (HAIs) represent a significant worldwide public health issue, continuing to contribute to increased healthcare costs, morbidity, and mortality despite substantial progress in infection control measures [1,2]. Despite advancements in infection control practices, HAIs continue to affect hospitalized patients, particularly those in intensive care units (ICUs). Among the most prevalent HAIs are surgical site infections (SSIs), ventilator-associated pneumonia (VAP), catheter-associated urinary tract infections (CAUTIs), and central line-associated bloodstream infections (CLABSIs) [3]. VAP caused by *Pseudomonas aeruginosa* is a major healthcare-associated infection in ICUs, associated with high morbidity, mortality, prolonged hospitalization, and significant antimicrobial resistance. It is recognized as one of the most common device-associated infections in critically ill patients admitted to ICUs [4].VAP is a subset of hospital-acquired pneumonia (HAP) that develops 48-72 hours or more following endotracheal intubation and the commencement of mechanical ventilation [5]. Pneumonia results from an inflammatory response triggered by the invasion of microorganisms into the typically sterile lower respiratory tract. The severity of this response depends on multiple factors, including the size of the inoculum, the virulence of the infecting organism, and the host’s immune status [6].

Gram-negative bacilli, particularly *P. aeruginosa* and *Acinetobacter *species, are among the principal microorganisms frequently isolated in cases of VAP [7]. Infections due to multidrug-resistant (MDR) pathogens, particularly *P. aeruginosa*, are considered high-risk and are associated with adverse clinical outcomes [8]. It is increasingly critical to distinguish between MDR strains, which exhibit non-susceptibility to at least one agent in three or more antimicrobial categories, and extensively drug-resistant (XDR) strains, which remain susceptible to only one or two antimicrobial categories [9]. Consequently, the increasing incidence of XDR *P. aeruginosa*, which demonstrates resistance against the majority of currently available antimicrobial agents, emerged as a major challenge in healthcare settings worldwide [9].

Despite the recognition of *P. aeruginosa* as a major etiological agent of VAP, variations in its reported prevalence and resistance patterns across different healthcare settings indicate significant regional and institutional differences in antimicrobial practices and infection control strategies [8,9]. This pathogenic success is driven by a sophisticated virulence arsenal; central to its ability to cause severe pneumonia is the Type III Secretion System (T3SS), a molecular syringe that delivers cytotoxic proteins directly into host cells, bypassing standard immune defenses. While several studies have highlighted its strong association with adverse clinical outcomes, including increased mortality and prolonged ICU stay, the extent of this impact is often influenced by the timeliness and appropriateness of antimicrobial therapy [8].

Furthermore, the emergence of MDR and XDR strains has complicated empirical treatment approaches, frequently resulting in delayed administration of effective antibiotics and poorer patient prognosis [9]. These observations underscore the necessity for continuous surveillance, region-specific antibiograms, and a more individualized therapeutic approach in managing *P. aeruginosa*-associated VAP. Effective management now requires the integration of robust antimicrobial stewardship to preserve drug efficacy and the use of rapid molecular diagnostics to identify specific resistance genes, such as blaVIM and blaNDM, early in the clinical course, as these genes are associated with carbapenem resistance and limited therapeutic options. In addition to its established role as a major nosocomial pathogen, *P. aeruginosa *has demonstrated a remarkable ability to adapt to the ICU environment through genetic plasticity and environmental persistence [8, 9].

Method

Search Strategy and Study Selection

The literature search primarily included studies published between 2000 and 2025, reflecting contemporary and clinically relevant research on VAP, particularly* P. aeruginosa*-associated VAP, antimicrobial resistance, virulence mechanisms, clinical outcomes, and emerging therapeutic strategies. Earlier landmark studies were also included, as these were considered scientifically important for understanding the evolution of antimicrobial resistance mechanisms and VAP pathogenesis. All selected studies were carefully evaluated based on their scientific relevance and contribution to the topic.

Studies eligible for inclusion were English-language articles focusing on VAP, especially those addressing *P. aeruginosa*, antimicrobial resistance mechanisms, MDR and XDR strains, virulence factors, diagnosis, prevention, and treatment strategies. Studies conducted in ICUs, hospital settings, and tertiary care centers were primarily considered. Original research articles, observational studies, clinical trials, systematic reviews, and review articles containing relevant microbiological and clinical data were included.

Studies were excluded if they focused exclusively on non-bacterial respiratory infections, lacked relevance to VAP or *P. aeruginosa*, did not report clinically relevant microbiological or antimicrobial susceptibility data, or consisted solely of case reports, editorials, letters to the editor, or conference abstracts lacking sufficient scientific detail. Duplicate publications were also excluded during the screening process. Study quality and potential sources of bias were evaluated during full-text review; however, a formal quantitative risk-of-bias assessment was not performed because of heterogeneity among the included studies.

**Figure 1:**
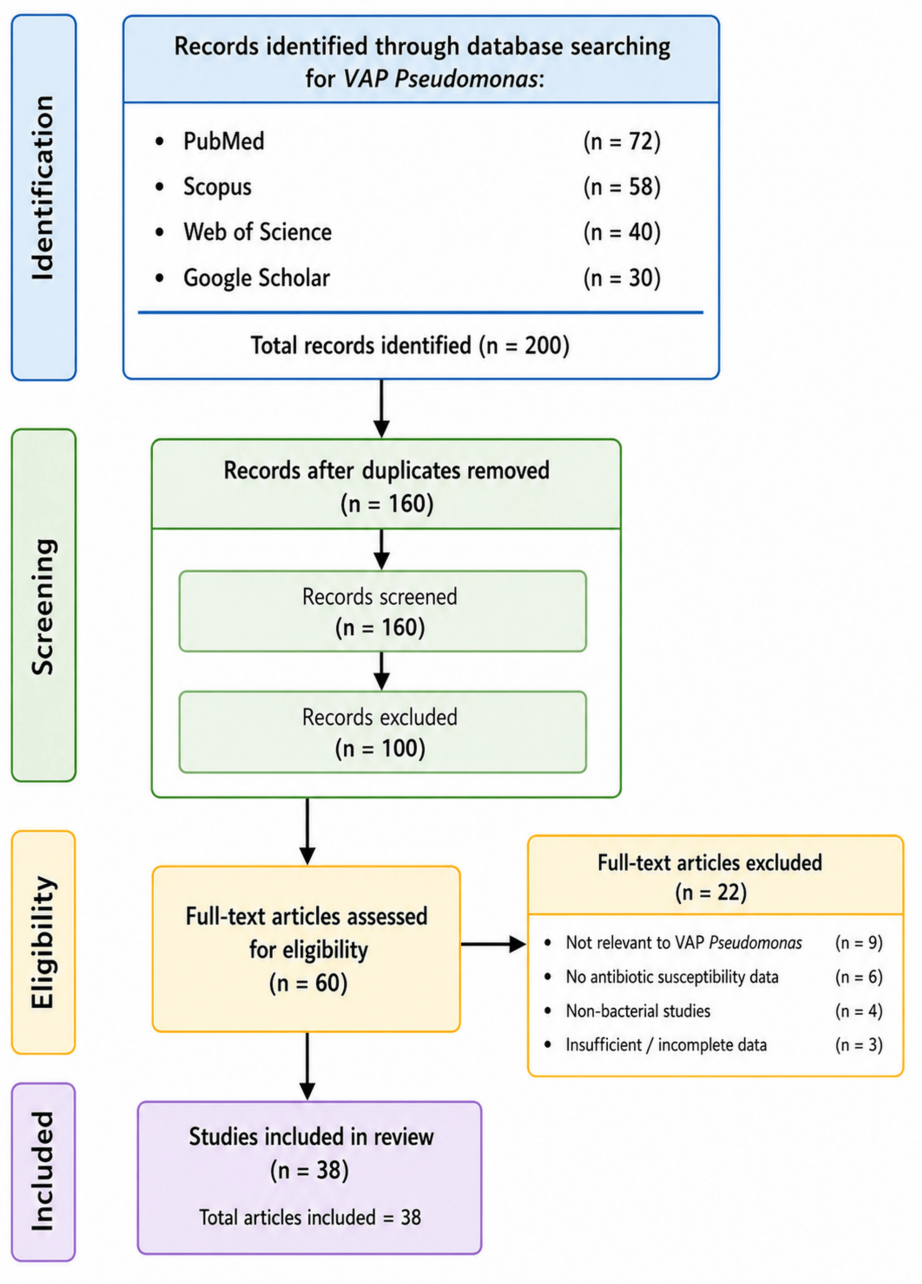
Article selection and screening process VAP: ventilator-associated pneumonia

Search strategies were individually adapted for each database to ensure comprehensive retrieval of relevant studies. For PubMed, the search strategy included the terms (“ventilator-associated pneumonia” OR “VAP”) AND (“*Pseudomonas aeruginosa*” OR “multidrug resistance” OR “antimicrobial resistance” OR “biofilm”). For Scopus, the search strategy used was TITLE-ABS-KEY (“ventilator-associated pneumonia” AND “*Pseudomonas aeruginosa*” AND “antibiotic resistance”). In Web of Science, the search was conducted using TS=(“ventilator-associated pneumonia” AND “Pseudomonas aeruginosa” AND “antimicrobial resistance”). For Google Scholar, the search terms used were “VAP Pseudomonas aeruginosa antimicrobial resistance biofilm ICU”. Boolean operators such as AND and OR were appropriately applied to optimize and refine the search results (Figure 1).

## Review

Healthcare-associated infections

HAIs are termed as infections that develop 48 hours or longer post-hospital admission or during the 30-day period following receipt of healthcare services. The term originally referred to infections acquired during hospitalization in acute-care settings, commonly described as nosocomial infections. Nonetheless, its usage has seen significant expansion to include infections occurring across a diverse range of healthcare environments, including outpatient clinics, ambulatory care centres, family medicine practices, long-term care facilities, and home-based healthcare settings [10,11]. These infections contribute substantially to prolonged hospital stays and adverse patient outcomes, emphasizing the ongoing importance of comprehensive surveillance and prevention approaches, and evidence-based clinical practices. Surgical site infections, VAP, catheter-associated urinary tract infections, and central line-associated bloodstream infections account for the predominant proportion of reported HAIs [3].

Ventilator-associated pneumonia

VAP is defined as a secondary pulmonary infection that develops 48-72 hours or later following endotracheal intubation. Clinically, it is characterized by the presence of a new or progressive pulmonary infiltrate on imaging, accompanied by systemic indicators of infection such as fever, leukocytosis or leukopenia, and purulent respiratory secretions. Beyond these clinical markers, the definitive diagnosis relies on the microbiological identification of a causative pathogen from lower respiratory tract samples [12]. VAP remains a significant burden in critical care, affecting approximately 8-20% of all ICU admissions; however, among patients requiring mechanical ventilation, the incidence significantly escalates to as high as 27% [13]. This high prevalence underscores the critical need for early detection and targeted antimicrobial intervention to mitigate the associated morbidity and mortality in these high-risk populations [12,13].

Pathogenesis and Mechanism of VAP

The primary pathogenic mechanisms of VAP are microaspiration of contaminated secretions and biofilm formation within the endotracheal or tracheal tube (TT). The presence of a TT suppresses normal upper airway defense reflexes, impairs effective coughing, and facilitates bacterial entry into the lower respiratory tract. During illness, hospitalization, and antibiotic therapy, aerobic gram-negative bacteria rapidly colonize the oropharynx. Contaminated secretions accumulate above the TT cuff and gradually migrate into the lower respiratory tract through folds in the cuff wall. Over time, antibiotic-resistant bacterial biofilms form on the inner surface of the tube, serving as a persistent source of infection. Ventilator cycling may further dislodge pathogen-rich biofilm fragments into the distal airways, contributing to pneumonia, particularly in critically ill and immunocompromised patients. The risk of developing VAP increases with prolonged duration of mechanical ventilation [14].

Beyond mechanical factors such as microaspiration, the pathogenesis of VAP is increasingly recognized as a multifactorial process involving complex host-pathogen interactions [6,14]. The ability of *P. aeruginosa *to form biofilms on endotracheal tubes not only facilitates persistent colonization but also significantly reduces antibiotic penetration, leading to suboptimal therapeutic responses [14].

At the molecular level, the transition from colonization to acute pneumonia is governed by the synchronized expression of virulence factors. The T3SS acts as a critical determinant of disease severity by injecting cytotoxic effector proteins, such as ExoU and ExoS, directly into the host’s alveolar epithelial cells and macrophages. While ExoU is a potent phospholipase that causes rapid cell lysis and necrosis, ExoS is a bifunctional protein that disrupts the actin cytoskeleton and inhibits phagocytosis, collectively subverting the host's innate immune signaling and creating a permissive environment for bacterial proliferation. This direct cellular assault not only causes acute tissue damage but also subverts the host's innate immune signaling, creating a permissive environment for the bacteria to transition from a planktonic state into the dense, antibiotic-recalcitrant biofilm structures characteristic of late-onset VAP [14].

In addition, critically ill patients often exhibit impaired immune responses, including reduced mucociliary clearance and altered inflammatory signaling, which further predispose them to infection [6]. These factors collectively contribute to the chronicity and severity of VAP, underscoring the need for therapeutic approaches targeting both microbial virulence and host susceptibility.

Although microaspiration and biofilm formation are well-established mechanisms in the pathogenesis of VAP, their relative contribution to disease progression may vary depending on patient-related and device-related factors [14]. The clinical significance of these mechanisms is underscored by the complex interplay between bacterial virulence factors and the host's dysregulated immune response, which often dictates the switch from acute infection to a chronic, biofilm-mediated state [6,14]. In particular, biofilm-associated infections are increasingly recognized as difficult to eradicate due to their inherent resistance to antimicrobial agents and host immune responses, thereby contributing to persistent infections and treatment failure [14]. Moreover, the interaction between bacterial virulence factors and host immune defenses plays a crucial role in determining disease severity, suggesting that VAP is not solely a consequence of bacterial invasion but also of dysregulated host responses [6]. This highlights the importance of targeting both microbial and host factors in the prevention and management of VAP.

Risk factors of VAP

The risk factors of VAP are listed in Table 1.

**Table 1:** Risk factors of VAP VAP: ventilator-associated pneumonia; COPD: chronic obstructive pulmonary disease; APACHE II: Acute Physiology and Chronic Health Evaluation II; SAPS II: Simplified Acute Physiology Score II; ICU: intensive care unit Reference: Ochoa et al., 2025 [15]

Patient-related factors	Treatment-related factors
Male sex	H₂ blocker (use may increase VAP risk by promoting gastric bacterial colonization and aspiration of contaminated gastric contents).
COPD	Tracheostomy
Impaired consciousness	Prior antibiotic use
Trauma (ICU admission)	Reintubation
Advanced age	Enteral feeding
Smoking	Nasogastric tube
Poisoning	Neuromuscular blockers
Diabetes mellitus	Prolonged intubation
Cardiac disease	Emergency intubation
Chronic kidney disease	Previous surgery
High APACHE II score	-
High SAPS II score	-
Corticosteroid use	-

Pathogens That Cause VAP

Table 2 shows the list of pathogens causing VAP.

**Table 2:** List of pathogens that cause VAP *Enterobacteriaceae: *Escherichia coli*, *Klebsiella pneumoniae*, *Proteus* spp., *Enterobacter* spp., *Serratia* spp., *Citrobacter* spp. VAP: ventilator-associated pneumonia; MSSA: methicillin-sensitive *Staphylococcus aureus*; MRSA: methicillin-resistant *Staphylococcus aureus*; ESBL: extended-spectrum beta-lactamase Reference: Kalanuria et al., 2014 [12]

Early-onset VAP	Late VAP
Streptococcus species	Pseudomonas aeruginosa
Hemophilus influenzae	Acinetobacter species
MSSA	MRSA
Enterobacteriaceae*	ESBL-producing bacteria

Major Agents of VAP

Non-fermentative gram-negative bacteria, particularly *Acinetobacter baumannii* and *P. aeruginosa*, are the predominant pathogens responsible for VAP [16]. These organisms are saprophytic in nature and broadly distributed across the environment [17]. The intrinsic and subsequently acquired antimicrobial resistance of these organisms, particularly against carbapenems, constitutes a major challenge in clinical practice [16]. Currently, carbapenems are considered among the most potent antimicrobial agents for the management of severe infections caused by *P. aeruginosa* and *A. baumannii*.

The occurrence of these major VAP agents and their contribution to MDR are noteworthy. In a culture-confirmed VAP study conducted among ICU patients, approximately 24.4% of isolates were identified as *P. aeruginosa*, which is frequently associated with plasmid-mediated metallo-beta-lactamase acquisition, efflux pump activation, and decreased expression of outer membrane porins. *Acinetobacter* species accounted for approximately 7.9% of isolates and were associated with metallo-beta-lactamase and carbapenemase production [12].

Pseudomonas from a historical perspective

The term “genera” was originally used by Ferdinand Cohn to describe bacteria with similar physical characteristics, while Migula formally defined the genus *Pseudomonas *in 1894. The name “aerobic pseudomonads” was later introduced by Stanier, Palleroni, and Doudoroff in 1972, who described them as single-celled, motile rods with polar flagella. Classification was further refined by incorporating G+C DNA content along with phenotypic characteristics [17].

Taxonomic Classification of Pseudomonas

The genus *Pseudomonas *belongs to the domain Bacteria, phylum Proteobacteria, class Gammaproteobacteria, order Pseudomonadales, and family *Pseudomonadaceae* [17].

Distribution of Species

Pseudomonads are aerobic, gram-negative, non-fermentative, non-spore-forming bacilli with polar flagella. Identification is based on biochemical properties, carbon assimilation patterns, and growth at 42°C. The genus is categorized into groups such as the fluorescent group, the Alcaligens group, and the Acidovarans group [17].

Pseudomonas aeruginosa

*P. aeruginosa* is a major pathogen involved in the pathophysiology of VAP. The increasing prevalence of MDR, XDR, and difficult-to-treat resistant (DTR) *P. aeruginosa* strains has significantly complicated therapeutic management and is associated with poorer clinical outcomes [18]. The pathogenicity of *P. aeruginosa* is largely mediated by QS systems. Specifically, the las and rhl QS systems produce signaling molecules known as autoinducers, which interact with target receptors to facilitate bacterial cell-to-cell communication and regulate virulence factor expression [19].

Multiple microbial virulence mechanisms contribute to the colonization of *P. aeruginosa*, facilitate evasion of host immune defenses, and promote infection. Adhesins facilitate attachment to host cells and surfaces. Through the T3SS, toxins are directly translocated into host cells, resulting in the disruption of cellular processes. Moreover, secreted proteins and enzymes contribute to tissue damage and help the pathogen evade the host immune response [19].

Medical importance of *P. aeruginosa*

*P. aeruginosa*, although a naturally occurring member of the *Pseudomonas *genus, is chiefly implicated in human infectious diseases [19]. Considered an opportunistic pathogen, it primarily causes nosocomial infections in immunocompromised individuals [19]. HAIs remain a significant contributor to morbidity and mortality [6]. Critically ill individuals, especially those admitted to ICUs, have lungs that are highly susceptible to infection.VAP is among the major healthcare-associated infections in ICUs, typically developing after at least 48 hours of mechanical ventilation [6]. Among the principal causative agents of nosocomial pneumonia, *P.* *aeruginosa *plays a key role, especially in intubated patients [9]. Infections caused by *P. aeruginosa* remain a serious concern, with reported mortality rates ranging from 40% to over 60% [16]. The occurrence of VAP is influenced by multiple determinants, notably prolonged mechanical ventilation, neurological disorders, trauma, sepsis, acute respiratory distress syndrome (ARDS), chronic lung disease, prior antibiotic use, and blood transfusions [13]. Moreover, *P. aeruginosa* is commonly recovered from hospital equipment, where its biofilm-forming ability promotes persistence and enhances transmission in healthcare environments [19].

Virulence factors

Multiple virulence determinants have been identified in *P. aeruginosa*. These may be synthesized by the bacterium itself or expressed on the bacterial cell surface [20].

Pili

Pili promote bacterial attachment to host cell surfaces, facilitate motility, and also promote biofilm formation. Five pilA alleles, designated groups I through V, have been identified in *P. aeruginosa* strains. Type IV pili (T4P) have garnered significant attention due to their role in mediating motility in the absence of flagella. Strains expressing T4P often exhibit "twitching motility," which is crucial for surface exploration and biofilm development [20].

Type I and II Secretion Systems

These contribute significantly to the virulence of *P. aeruginosa*. The type I secretion system (T1SS) releases toxins into extracellular areas in a single step. Alkaline protease, one of the most studied T1SS toxins, inhibits fibrin synthesis and facilitates bacterial spread. Other toxins secreted through these systems, including exotoxin A, phospholipase C, protease IV, and elastase, contribute to cytotoxicity, tissue destruction, inflammatory responses, and bacterial colonization. These virulence factors are associated with severe pneumonia, persistent lung injury, treatment failure, prolonged mechanical ventilation, and increased mortality in patients with VAP [20].

T3SS

The complex T3SS injects exotoxins directly into the cytoplasm of host cells. Four key exotoxins contribute to pathogenicity: ExoU, a phospholipase, induces necrosis of phagocytes and parenchymal cells, promoting apoptosis; ExoY, an adenylate cyclase, disrupts pulmonary endothelial barrier integrity; and ExoT and ExoS, bifunctional proteins, impair cell growth by inhibiting DNA synthesis and altering the cytoskeleton, thereby affecting adhesion. Clinical outcomes are frequently more severe in infections caused by *P. aeruginosa* strains expressing T3SS [20].

QS Molecules

QS enables cell-to-cell communication through signaling molecules known as autoinducers. Detection of these autoinducers by specific receptors triggers alterations in gene expression. *P. aeruginosa* possesses three QS systems: two LuxI/LuxR-type circuits and the *Pseudomonas *quinolone signal (PQS) system, which regulates the production of virulence factors, including elastase, exotoxin A, and proteases, as well as biofilm formation [20].

Other Virulence Factors

Lipopolysaccharide (endotoxin) on the bacterial outer membrane prevents effective host defense. Pyoverdin, a siderophore, competes with host proteins for iron. Pyocyanin inhibits ciliary function, suppresses lymphocyte proliferation, and induces the formation of reactive oxygen species, which damage tissue. Alginate, an extracellular polymer, resists opsonization and phagocytosis, scavenges free radicals from macrophages, and impairs complement activation and neutrophil chemotaxis [20].

Laboratory diagnosis of *P. aeruginosa*

*P. aeruginosa* grows readily on routine laboratory media, including nutrient agar, blood agar, and MacConkey agar. Clinical isolates frequently exhibit β-haemolysis when cultured on blood agar. Selective media containing selective agents, including cetrimide and Irgasan, are especially useful for isolating *P. aeruginosa* from specimens harbouring diverse microbial populations. The organism is non-fermentative, oxidase positive, and negative for indole and hydrogen sulphide production. It also shows negative reactions in the methyl red and Voges-Proskauer tests. Additional biochemical characteristics, including gelatin liquefaction, acetamide hydrolysis, and nitrate reduction to nitrogen gas, further aid in identification, particularly when pyocyanin production is absent or inconclusive [21].

Mechanisms of antimicrobial resistance in *P. aeruginosa*

The antimicrobial resistance exhibited by *P. aeruginosa* is multifactorial and dynamic, making it one of the most challenging pathogens to treat in clinical practice. While intrinsic resistance mechanisms provide a baseline level of reduced susceptibility, the acquisition of additional resistance determinants through horizontal gene transfer significantly enhances their adaptive potential in hospital environments [21].

The resistance profile of* P. aeruginosa* is not merely a collection of isolated traits but rather a complex interplay between intrinsic, acquired, and adaptive mechanisms that often coexist within a single isolate [21]. For instance, while intrinsic factors like the MexAB-OprM efflux system provide an immediate defense against various antibiotic classes, they work in tandem with adaptive biofilm formation to create a highly shielded environment. Within the biofilm matrix, persister cells exhibit altered metabolic activity and reduced growth rates, rendering them nearly impervious to traditional bactericidal agents that target active cell division [14,21].

Clinical studies have demonstrated that infections caused by MDR strains are associated with higher rates of treatment failure and mortality, largely due to limited therapeutic options and delayed initiation of appropriate therapy [22]. Furthermore, adaptive resistance mechanisms, particularly biofilm formation, contribute to chronic and recurrent infections by enabling bacterial persistence even in the presence of antimicrobial agents [14,21]. These factors collectively emphasize the urgent need for novel therapeutic strategies and improved antimicrobial stewardship.

The resistance profile of *P. aeruginosa* is further complicated by the interplay between intrinsic, acquired, and adaptive mechanisms, which often coexist within the same strain [21]. Intrinsic resistance is primarily attributed to low outer membrane permeability and the presence of efflux pump systems, which actively expel antimicrobial agents from the bacterial cell [21]. Acquired resistance frequently arises through horizontal gene transfer, including plasmid-mediated acquisition of β-lactamase enzymes such as metallo-β-lactamases, which confer resistance to carbapenems [12,21]. 

This multifaceted resistance pattern is further exacerbated by the compounding cycle of ICU management, where the selective pressure of broad-spectrum antibiotic use drives the over-expression of chromosomal AmpC β-lactamases and the strategic down-regulation or loss of OprD porins. This synergy significantly narrows the window of effective treatment, often leading to the emergence of XDR phenotypes during the course of therapy [21,23]. In addition to these mechanisms, adaptive resistance plays a crucial role during infection, particularly in biofilm-associated states. Within biofilms, bacterial cells exhibit altered metabolic activity and reduced growth rates, rendering them less susceptible to antibiotics that target actively dividing cells [14]. This dynamic and multifaceted resistance pattern significantly limits therapeutic options and contributes to treatment failure in critically ill patients [21]. Collectively, these layered defense strategies necessitate a shift toward personalized therapy based on rapid molecular resistance profiling rather than traditional empirical approaches. The mechanism of antimicrobial resistance in *P. aeruginosa* is shown in Table 3.

**Table 3:** Mechanisms of antimicrobial resistance in P. aeruginosa. AmpC: chromosomal class C beta-lactamase; OprD: outer membrane porin D; MexAB-OprM: multidrug efflux system; ICU: intensive care unit Reference: Amankwah et al., 2022 [21].

Resistance Category	Description	Major Mechanisms	Clinical Relevance
Intrinsic Resistance	Natural resistance.	Low permeability, Efflux pumps, AmpC.	Reduced baseline susceptibility Carbapenem resistance (OprD loss).
Acquired Resistance	Genetic changes.	Gene mutations, Plasmid-mediated resistance, Enzyme production.	High-level resistance Treatment failure risk.
Adaptive Resistance	Temporary response	Biofilm formation, persister cells Stress response.	Chronic infection Reduced antibiotic efficacy.

Genotypic determinants and high-risk clones 

The clinical significance of *P. aeruginosa* in the ICU is profoundly influenced by its genetic plasticity and the resulting impact on patient survival. Crucially, the presence of acquired resistance determinants serves as an independent predictor of treatment failure and increased mortality, as they significantly limit the window of effective empirical therapy [23]. Furthermore, the synergistic interplay between these genetic markers and the bacterial stress response ensures the persistence of the pathogen even under aggressive antimicrobial challenge, often leading to recurrent infections and chronic colonization in critically ill patients [21].

This molecular landscape is increasingly defined by specific genotypic markers that drive the dissemination of MDR. High-risk international clones, most notably ST235, ST111, and ST175, have emerged as dominant lineages in hospital settings, often carrying a heavy burden of mobile genetic elements such as integrons and transposons. The acquisition of carbapenemase genes, particularly the metallo-beta-lactamases (MBLs) such as bla_VIM_, bla_IMP_, and bla_NDM_, is of paramount clinical concern; these genotypes confer resistance to nearly all traditional beta-lactams and even many newer beta-lactam/inhibitor combinations [23]. Additionally, the genetic regulation of the oprD porin and the over-expression of efflux systems like MexAB-OprM provide a robust genetic basis for the rapid development of XDR phenotypes under the selective pressure of ICU therapy [21].

Clinical outcomes, mortality, and guidelines

HAIs and VAP caused by P. aeruginosa are associated with significant adverse clinical outcomes, including increased mortality, prolonged ICU stay, and extended duration of mechanical ventilation. A large multicenter study demonstrated that *P. aeruginosa* nosocomial pneumonia is linked with high mortality rates and substantial clinical burden, particularly in critically ill patients [22]. The presence of MDR strains further exacerbates outcomes, as these infections are associated with delayed initiation of appropriate antimicrobial therapy and increased risk of treatment failure [23]. In addition, MDR *P. aeruginosa* infections have been identified as an independent predictor of mortality, emphasizing the importance of early and effective therapeutic intervention [23].

The clinical impact of *P. aeruginosa*-associated VAP is further intensified in cases involving MDR strains. While the crude mortality rate for these infections can exceed 40%, the attributable mortality-deaths directly resulting from the infection itself-is estimated to range between 13% and 30%. This distinction underscores that *P. aeruginosa *remains a high-risk pathogen even with intervention, as its presence significantly increases the probability of a fatal outcome compared to VAP caused by less virulent organisms. Studies have demonstrated that infections caused by MDR organisms are associated with delayed administration of appropriate antimicrobial therapy, which is a key determinant of increased mortality [22,23]. Moreover, prolonged ICU stays and extended durations of mechanical ventilation not only increase healthcare costs but also predispose patients to secondary infections and complications [22]. This 'deleterious loop' involving persistent colonization and long-term mechanical ventilation not only places a heavy burden on healthcare systems but also markedly reduces the functional recovery and future quality of life for surviving patients [22,23]. These findings reinforce the importance of early diagnosis and timely initiation of effective antimicrobial therapy in improving patient outcomes.

Current clinical practice guidelines recommend the use of empiric broad-spectrum antimicrobial therapy in patients with suspected VAP, followed by de-escalation based on microbiological findings and antimicrobial susceptibility testing [24]. The logic underlying this strategy is to ensure the rapid administration of therapy effective against highly resistant strains, while simultaneously practicing de-escalation to reduce the selective evolutionary pressure that promotes further antimicrobial resistance [24,25]. These guidelines also emphasize the importance of local antibiograms in guiding therapy and recommend combination therapy in patients at high risk for MDR pathogens [24]. Early initiation of appropriate antibiotics, along with adherence to evidence-based management strategies, has been shown to significantly improve patient outcomes and reduce mortality in VAP [24].

VAP treatment

Table 4 demonstrates the treatment of VAP.

**Table 4:** Treatment of VAP VAP: ventilator-associated pneumonia; HAP: hospital-acquired pneumonia; MDR: multidrug-resistant References: [24-28]

Antimicrobial	Characteristics	Use
Piperacillin-tazobactam	Broad Gram-negative coverage.	Early VAP, low MDR risk.
Meropenem	Covers MDR organisms.	Severe infections, high MDR risk.
Ceftolozane/tazobactam	Strong anti-pseudomonal activity.	MDR *P. aeruginosa*.
Ceftazidime/avibactam	Active vs carbapenemase producers.	Resistant Enterobacteriaceae.

Antimicrobial selection should be guided by local antibiograms and antimicrobial susceptibility testing. In selected clinical scenarios, monotherapy with antipseudomonal agents may be adequate; however, combination therapy is generally preferred in patients with a high likelihood of MDR infections to ensure appropriate empirical coverage [24]. This approach aims to broaden the initial spectrum and exploit potential synergistic effects between different classes, such as beta-lactams and aminoglycosides or polymyxins, thereby increasing the probability of at least one agent being active against highly resistant isolates [24,26]. At the same time, the use of broad-spectrum antibiotics should be carefully optimized to avoid unnecessary selective pressure that may accelerate resistance development, highlighting the critical role of antimicrobial stewardship programs [24,25].

Recent clinical studies evaluating newer antimicrobial agents, including ceftolozane-tazobactam and ceftazidime-avibactam, have reported outcomes comparable to or better than conventional therapies in managing resistant infections, although the emergence of resistance during therapy remains a significant concern [26,29,30]. Of particular note is cefiderocol, a novel siderophore cephalosporin that utilizes a unique "Trojan horse" mechanism. By binding to extracellular free iron and being actively transported across the outer membrane via bacterial iron-uptake transporters, cefiderocol achieves high periplasmic concentrations even in the presence of porin mutations or efflux pump upregulation, making it a critical last-resort option for carbapenem-resistant *P.* *aeruginosa* [27,31,32]. These observations underscore the importance of tailoring treatment regimens based on local antibiogram data and individual patient factors.

Although several antipseudomonal agents, including β-lactam/β-lactamase inhibitor combinations and carbapenems, remain the cornerstone of therapy for VAP, their effectiveness is increasingly threatened by the rapid emergence of resistance [25,26]. Comparative clinical trials have demonstrated that newer agents such as ceftolozane-tazobactam may offer improved outcomes in infections caused by resistant strains; however, their widespread use may also contribute to the development of further resistance if not carefully regulated [26]. The pharmacological optimization of these agents, such as using extended or continuous infusion strategies, is increasingly recommended to maximize the time the free drug concentration remains above the minimum inhibitory concentration (fT > MIC), which is essential for achieving microbiological eradication in the hyperdynamic state of critically ill ICU patients [24,33]. In addition, variability in regional resistance patterns necessitates the use of local antibiograms to guide empirical therapy and optimize treatment outcomes [24]. Therefore, a balanced approach incorporating early appropriate therapy, timely de-escalation, and antimicrobial stewardship is essential to improve clinical outcomes in patients with *P. aeruginosa *VAP [24,25].

Emerging therapies in VAP management

Recent advances in the management of *P. aeruginosa* infections have focused on the development of novel antimicrobial agents and alternative therapeutic strategies to overcome rising resistance. Ceftolozane-tazobactam has demonstrated favorable clinical and microbiological outcomes in infections caused by MDR and XDR strains, although emerging resistance during therapy remains a concern [29]. 

Cefiderocol represents an important therapeutic option for carbapenem-resistant and DTR *P. aeruginosa* isolates, particularly in critically ill patients with limited treatment alternatives [31,32]. It is a siderophore cephalosporin that utilizes an iron-mediated bacterial entry mechanism, commonly described as a "Trojan Horse" strategy [31]. The agent has demonstrated strong in vitro activity, with susceptibility rates exceeding those of several other β-lactam/β-lactamase inhibitor combinations [32]. In the APEKS-NP (Acinetobacter, Pseudomonas, Escherichia coli, Klebsiella, Stenotrophomonas - Nosocomial Pneumonia) randomized, double-blind, phase 3 trial, cefiderocol demonstrated clinical efficacy comparable to high-dose extended-infusion meropenem in gram-negative nosocomial pneumonia [27]. However, clinical evidence regarding its long-term effectiveness in severe VAP remains limited, and the emergence of resistance during therapy has been reported [30]. In addition, accurate cefiderocol susceptibility testing requires specialized laboratory methodologies that may not be universally available. Therefore, cefiderocol should be used judiciously within antimicrobial stewardship programs and preferably guided by susceptibility testing results and local resistance epidemiology.

Despite these advances, increasing resistance to last-resort agents such as polymyxins has been reported, further limiting available treatment options and highlighting the dynamic nature of antimicrobial resistance in clinical settings [30]. Although several newer antimicrobial agents have demonstrated strong in vitro efficacy, their clinical use is often constrained by challenges such as high cost, limited availability, and the risk of rapid development of resistance during therapy [29,31,34].

As resistance to "last-resort" agents continues to be reported, alternative therapeutic modalities are being explored [30]. Bacteriophage therapy is being investigated as a key approach to overcome the limitations of conventional antimicrobial treatment [35,36]. Early clinical case experience, such as the first 10 consecutive cases of intravenous phage therapy at a single center, provides critical lessons for treating MDR infections [35]. Furthermore, alternative treatment modalities, including quorum-sensing inhibitors, remain largely investigational and require further validation through well-designed, large-scale clinical studies. These non-traditional approaches aim to attenuate bacterial virulence without imposing the same selective pressure as conventional bactericidal agents, potentially slowing the evolution of resistance.

In addition, growing interest in biofilm-targeted therapies, including nanoparticle-based drug delivery systems and anti-biofilm agents, reflects the need to address persistent infections associated with biofilm formation on medical devices [32]. Collectively, these findings emphasize the importance of integrating novel therapeutics with antimicrobial stewardship and surveillance strategies to improve clinical outcomes in *P. aeruginosa*-associated VAP [33,34].

Recent advances in diagnosis and management

Despite the availability of current antimicrobial therapies, emerging resistance patterns and evolving diagnostic approaches necessitate continuous advancements in the management of VAP. Recent advances in the management of *P. aeruginosa*-associated VAP reflect evolving epidemiological trends, particularly the increasing prevalence of MDR and XDR strains, with a higher burden reported in low- and middle-income countries compared to developed regions [34]. These trends have necessitated the development of novel therapeutic agents, among which ceftolozane-tazobactam and cefiderocol have demonstrated promising activity against resistant isolates, although the emergence of resistance during therapy remains a concern [29,31].

In addition, alternative approaches such as bacteriophage therapy and anti-QS agents are being explored to overcome the limitations of conventional antimicrobial treatment [35,36]. Bacteriophage therapy, utilizing viral agents to specifically lyse *P.* *aeruginosa*, offers a targeted approach that minimizes damage to the host microbiome, while QS inhibitors aim to disarm the bacteria by disrupting cell-to-cell signaling, thereby preventing the expression of virulence factors and biofilm maturation without imposing the same selective pressure as bactericidal drugs [35,36].

Parallel advancements in diagnostic technologies have significantly improved early pathogen detection and resistance profiling. Molecular methods, including polymerase chain reaction (PCR) based assays, along with rapid detection of antimicrobial resistance genes, enable timely identification of pathogens and resistance determinants, facilitating targeted therapy [37]. Multiplex PCR platforms now allow for the simultaneous detection of high-risk carbapenemase genes, such as bla_VIM_, bla_IMP_, and bla_NDM_, which are frequently implicated in XDR *P. aeruginosa *outbreaks in the ICU [37]. Furthermore, rapid identification techniques such as matrix-assisted laser desorption ionization-time of flight mass spectrometry (MALDI-TOF MS) enhance diagnostic accuracy and reduce turnaround time, thereby improving clinical decision-making and patient outcomes. MALDI-TOF MS utilizes protein fingerprinting to identify *P. aeruginosa* isolates directly from positive culture plates within minutes, significantly narrowing the time between clinical suspicion and the initiation of effective, pathogen-directed therapy [38].

VAP prevention

Care Bundles

Care bundles are groups of evidence-based interventions for a specific condition that, when implemented together, significantly improve patient outcomes [14].

TT Modification

VAP prevention strategies have focused on TT cuff design, as the cuff can provide a continuous pathway between the oral cavity and distal airways, facilitating microaspiration [14].

Cuff Pressure Control

Maintaining cuff pressure at 20-30 cmH₂O is critical. Pressures below 20 cmH₂O may allow secretions to flow along the tracheal wall, while pressures above 30 cmH₂O can damage the mucosa. Despite careful adjustments, cuff pressure often fluctuates. Various approaches have been implemented to maintain and continuously monitor endotracheal tube cuff pressure [14].

Subglottic Secretion Drainage

Devices for subglottic secretion drainage include a suction source and an additional port above the TT cuff, allowing continuous or intermittent removal of secretions. This intervention reduces the period of mechanical ventilation, time to VAP onset, and ICU length of stay [14].

TT Cuff Design

Conventional TT cuffs are high-volume, low-pressure, and made of polyvinyl chloride. When inflated, they may fold, creating channels for secretion passage. Tapered cuffs made of ultra-thin polyurethane provide better protection against secretion leakage and VAP development [14].

TT Coating

Coating the TT interior with antimicrobial agents, such as silver, chlorhexidine, or titanium dioxide, can prevent bacterial colonization and biofilm formation [14].

Nebulized Gentamicin

It has been investigated as an adjunctive strategy to achieve higher local antibiotic concentrations within the TT and reduce biofilm formation. However, aerosolized antibiotics are not routinely recommended as universal VAP prevention measures because current evidence remains limited, and concerns exist regarding antimicrobial resistance, bronchospasm, and inconsistent drug delivery [14].

Kinetic Therapy

Mechanical rotation of patients by 40° improves mucociliary clearance compared with conventional two-hourly turns. Although it lowers VAP incidence, it does not affect ventilation duration, ICU stay, or mortality, and may result in complications such as arrhythmias or accidental extubation [14].

Maintenance of Airway Equipment

Biofilm formation on the TT begins within 24 hours of intubation. Measures such as closed-circuit suctioning, heat and moisture exchangers, strict hand hygiene, and changing tubes only when necessary can reduce biofilm development [14].

Feeding

While early enteral feeding is beneficial in critical care, post-pyloric feeding may reduce VAP incidence by minimizing reflux and aspiration of gastric contents [14].

Probiotics

Administration of probiotics enhances microbial balance in the oropharynx and stomach, reducing VAP prevalence without affecting ventilation duration or mortality [14].

Intubation-Related Events

Reducing the risk of VAP can also be achieved by avoiding unplanned extubations, minimizing re-intubation, and shortening intubation duration using sedative holds and structured weaning strategies [14].

Antimicrobial stewardship and clinical implications

Antimicrobial stewardship plays a pivotal role in controlling the emergence and spread of MDR *P. aeruginosa* in ICU settings. Strategies such as early initiation of appropriate empirical therapy followed by de-escalation based on microbiological results have been shown to optimize clinical outcomes while minimizing unnecessary antibiotic exposure. The use of local antibiograms is essential in guiding empirical treatment decisions, particularly in regions with high resistance rates [24]. In addition, antibiotic cycling and rotation strategies in ICUs have been proposed to reduce selective pressure, although their effectiveness remains variable across studies [33]. Implementing these stewardship interventions, along with strict infection control practices, is crucial for preserving the efficacy of existing antimicrobial agents and improving patient outcomes in VAP.

## Conclusions

VAP remains a significant healthcare-associated infection, particularly among critically ill patients requiring mechanical ventilation. Among the causative pathogens, *P. aeruginosa* continues to pose a major clinical challenge due to its extensive virulence mechanisms, biofilm-forming ability, and rapidly evolving antimicrobial resistance. The increasing prevalence of MDR and XDR strains has further complicated therapeutic decision-making and is associated with higher mortality, prolonged ICU stay, and increased healthcare burden. Despite advances in antimicrobial therapy, including newer β-lactam/β-lactamase inhibitor combinations and novel agents such as cefiderocol, treatment outcomes remain suboptimal in resistant infections. Preventive strategies, including ventilator care bundles, improved endotracheal tube design, and strict infection control practices, remain essential in reducing VAP incidence.

Future efforts should focus on strengthening antimicrobial stewardship, integrating rapid molecular diagnostics for early targeted therapy, and developing innovative treatment approaches such as bacteriophage therapy, anti-biofilm agents, and immunotherapeutic strategies. A multidisciplinary and evidence-based approach will be crucial to effectively combat *P. aeruginosa*-associated VAP and improve patient outcomes in critical care settings.
